# Prevalence and risk factors for gastrointestinal parasites in small-scale pig enterprises in Central and Eastern Uganda

**DOI:** 10.1007/s00436-016-5296-7

**Published:** 2016-10-26

**Authors:** Kristina Roesel, Ian Dohoo, Maximilian Baumann, Michel Dione, Delia Grace, Peter-Henning Clausen

**Affiliations:** 1 Freie Universität Berlin, Institute for Parasitology and Tropical Veterinary Medicine, Robert-von-Ostertag-Str. 7-13, 14163 Berlin, Germany; 2 International Livestock Research Institute, P.O. Box 30907, Nairobi, 00100 Kenya; 3 University of Prince Edward Islands, Charlottetown, PEI C1A 4P3 Canada; 4 Freie Universität Berlin, FAO Reference Centre for Veterinary Public Health, Koenigsweg 67, 14163 Berlin, Germany; 5 International Livestock Research Institute, P.O. Box 24384, Kampala, Uganda

**Keywords:** Gastrointestinal helminths, Coccidia, Pigs, Husbandry practices, Risk factors, Uganda

## Abstract

In Eastern Africa, small-scale pig keeping has emerged as a popular activity to generate additional household income. Infections of pigs with gastrointestinal helminths can limit production output, increase production costs, and pose zoonotic risks. A cross-sectional, community-based study in three districts in Eastern and Central Uganda examined the prevalence of gastrointestinal helminthes and associated risk factors in 932 randomly sampled pigs. Using the combined sedimentation-flotation method, 61.4 % (58.2–64.5 %, 95 % confidence interval [CI]) tested positive for one or more gastrointestinal helminths, namely, strongyles (57.1 %, 95 % CI), *Metastrongylus* spp. (7.6 %, 95 % CI), *Ascaris suum* (5.9 %, 95 % CI), *Strongyloides ransomi* (4.2 %, 95 % CI), and *Trichuris suis* (3.4 %, 95 % CI). Coccidia oocysts were found in 40.7 % of all pigs sampled (37.5–44.0 %, 95 % CI). Significant differences across the three districts were observed for the presence of *A. suum* (*p* < 0.001), *Metastrongylus* spp. (*p* = 0.001), *S. ransomi* (*p* = 0.002), and coccidia oocysts (*p* = 0.05). All animals tested negative for *Fasciola* spp. and *Balantidium coli*. Thirty-five variables were included in univariable analyses with helminth infection as the outcome of interest. A causal model was generated to identify relationships among the potential predictors, and consequently, seven variables with *p* ≤ 0.15 were included in a multivariable analysis for helminth infection. The final regression models showed that routine management factors had a greater impact on the prevalence of infection than regular, preventive medical treatment or the level of confinement. Factors that negatively correlated with gastrointestinal infection were the routine removal of manure and litter from pig pens (*p* ≤ 0.05, odds ratio [OR] = 0.667) and the routine use of disinfectants (*p* ≤ 0.05, OR = 0.548).

**Electronic supplementary material:**

The online version of this article (doi:10.1007/s00436-016-5296-7) contains supplementary material, which is available to authorized users.

## Introduction

Pig keeping is an important livelihood activity for farmers in Eastern and Southern Africa (Phiri et al. 2003; Mutua et al. 2011). In Uganda, traditionally a cattle-keeping community, small-scale pig keeping has grown rapidly since the 1980s; its main objective is income generation (Muhanguzi et al. 2012; Ouma et al. 2014). Pigs grow fast, have high fecundity rates and short generation intervals resulting in quick generation of cash for farmers; women prefer to rear pigs as they do not require as much physical labor in handling, and pig keeping needs less land (ILRI 2011). Most of the pigs in Uganda are produced under traditional smallholder systems, which are often considered wasteful and not as profitable as intensive production systems due to poor feed conversion, high mortality rates, low reproductive rates, and poor final products (Lekule and Kyvsgaard 2003). However, for resource-poor farmers, the traditional pig production system is attractive (Verhulst 1993; Phiri et al. 2003) because it requires much less space (Delgado et al. 2001) and little to no housing due to the pig’s natural scavenging behavior to utilize kitchen leftovers and agricultural waste (Lekule and Kyvsgaard 2003). On the other hand, this scavenging behavior exposes them to diseases such as African swine fever or zoonotic agents such as *Taenia solium*, which have been reported from Uganda (Phiri et al. 2003; Thomas et al. 2013; Atuhaire et al. 2014; Kungu et al. 2016).

One of the biggest constraints to pig confinement is the cost of feed which usually accounts for up to 80 % of all costs in intensive pig production (Verhulst 1993; Mutua et al. 2011; Muhanguzi et al. 2012). Infections with gastrointestinal parasites may reduce production as they potentially cause lower average daily gains (ADGs) and may also result in poorer feed conversion ratios (Hale and Stewart 1979; Hale et al. 1985). In Central and Eastern Uganda, African swine fever and worms are considered the most important disease constraints by smallholder pig farmers (Muhanguzi et al. 2012; Dione et al. 2014). The objectives of the reported survey were (a) to estimate the prevalence of pig infection with common intestinal parasites in Central and Eastern Uganda, (b) to assess risk factors that are associated with the prevalence of parasites, and (c) to improve the evidence base for developing recommendations on gastrointestinal parasite management in smallholder pig production systems in the tropics.

## Materials and methods

### Study area

From April to July 2013, towards the end of the rainy season, a cross-sectional survey was conducted in Masaka, Mukono, and Kamuli Districts in Central and Eastern Uganda. Uganda’s climate is equatorial but temperatures and precipitation levels vary across the country depending on the altitude of the region and the proximity to the lake. Study districts were located in the lowland areas and at an average altitude of 1100 m above sea level. Of the three districts, Kamuli has the highest poverty levels (Ochola 2012) and lowest pig density (approximately 36 pigs per km^2^), while Masaka has the highest pig density in the country (108 pigs per km^2^), followed by Mukono (42 pigs per km^2^).^1^

### Site selection

**Fig. 1:**
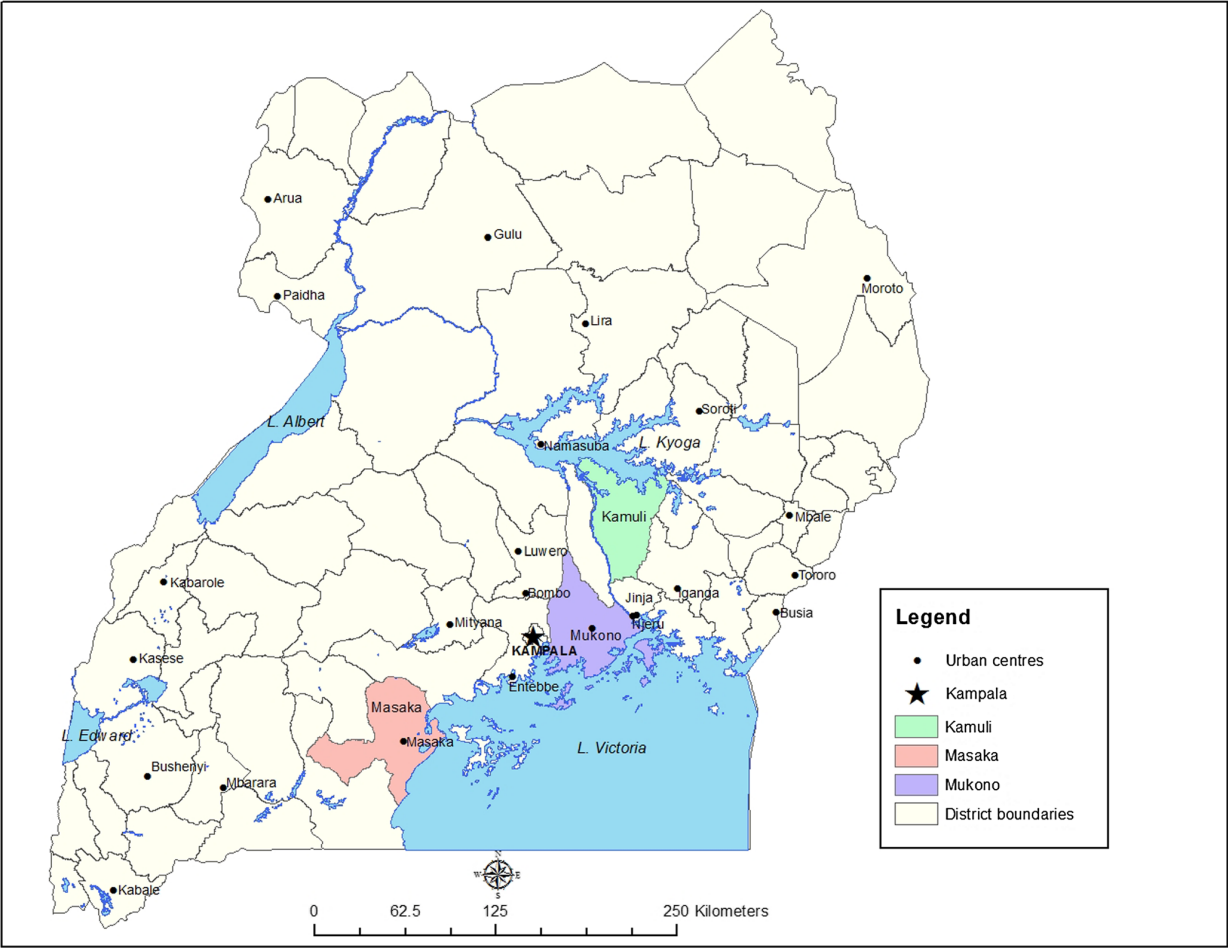
Selected sites for pig farm sampling in Kamuli, Masaka, and Mukono Districts of Central and Eastern Uganda (April–July 2013) (ILRI/Pamela Ochungo)

Kamuli, Mukono, and Masaka Districts were selected for an initial assessment of constraints and opportunities in smallholder pig production in Uganda by the Smallholder Pig Value Chain Development project led by the International Livestock Research Institute (ILRI). The detailed site selection process is described elsewhere (Dione et al. 2014; Ouma et al. 2014). For each district, four to six sub-counties with a high pig population, based on the 2008 livestock census (MAAIF/UBOS 2009), were purposively selected for further categorizing villages into value chain domains. These were broadly classified by the locality of production and consumption as follows: rural production for rural consumption (rural-rural [RR]), rural production for urban consumption (rural-urban [RU]), and urban production for urban consumption (urban-urban [UU]) (Ouma et al. 2014). For each district, two sub-counties were purposively selected to represent each value chain domain type. Within each selected sub-county, two to three villages were randomly selected, eventually totaling 35 villages for initial scoping and group discussions (Ouma et al. 2014). For the present prevalence estimate survey, 21 villages out of the 35 were purposively selected across the 3 districts, based on financial and logistic resources (Fig. 1).

### Sample size calculation

The original sample size was calculated to estimate district-level prevalence and considering an infinite population using *n* = [*Z* ^2^ *P*(1 − *P*)]/*d* ^2^, where *n* is the required sample size; *Z* is the multiplier from a standard normal distribution (1.96) at a probability level of 0.05; *P* is the estimated prevalence which is most conservatively estimated to be 50 %, considering that there were no reference data from pigs in the area under study; (1 − *P*) is the probability of having no disease; and *d* was the desired precision level (5 %). Therefore, a sample size of 384 pigs per district was required for the study. To increase precision, a sample size of 400 pigs in each district was planned.

### Selection of the pigs

A list of all pig-keeping households in the selected villages was generated with local partners. Households invited to participate in the study were randomly selected from that list. One animal per household was selected for collection of feces if it was 3 months or older, not weak or emaciated, not pregnant, or with a litter less than 2 months old.

### Collection of samples and metadata

Fecal samples were collected from the rectum of the pig unless it had just defecated, in which case fresh manure was collected from the floor of the pen or the spot where the pig had been tethered by the owner. Samples were placed in BD Falcon^™^ 50-ml conical tubes and labeled with the household identifier number. They were stored in a cold box and transported to the field laboratory, where the identification numbers were cross-checked and the samples were stored overnight in the cold box for processing the following morning. Information on the sampled pig (biodata) such as the clinically apparent health status, age, breed, rectal temperature, and last parasite treatment was recorded. A structured questionnaire on self-reported biosecurity and husbandry practices was administered to the owner of the pigs.

### Combined sedimentation-flotation method

The fecal samples were prepared for microscopy by means of the combined sedimentation-flotation method (Eckert et al. 2008), a qualitative test for the detection of trematode and nematode eggs, coccidia oocysts, and protozoan cysts (e.g., *Balantidium coli*). A lump of fecal matter the size of a walnut was thoroughly mixed with 50-ml tap water in a petri dish and poured into a 250-ml beaker through a tea strainer (mesh size 500–800 μm) to separate large particles. Additional tap water was added with a washing bottle to rinse the mesh. The tea strainer was left on top of the beaker to allow the liquid to strain through. After 30 min, the supernatant was discarded carefully and without interruption. The sediment was swirled and 2 ml was poured into a 15-ml BD Falcon^™^ centrifuge tube. The remains were left in the beaker for microscopic examination. A saturated salt solution was prepared by dissolving 400 g kitchen salt in 1000 ml water; 500 g sugar was added to the salt solution and stirred until the sugar was dissolved. The centrifuge tube was filled with the sugar-salt solution up to the 14-ml mark and centrifuged at 300×*g* for 5 min. After centrifugation, the material on the surface was transferred to a microscope slide using a bent inoculation loop, covered with a cover slip, and examined at ×400 magnification. Due to their specific gravity, parasitic stages first sediment in water and subsequently float in a solution of higher specific gravity (1.280). Particles in the sample, including trematode eggs, will sink to the bottom. Therefore, the remaining sediment from the beaker was diluted again with tap water and left again for 3 min to allow trematode eggs to sediment. Subsequently, the supernatant was discarded; the beaker was swirled and filled up with water again. After another 3 min, the supernatant was discarded again and the sediment was transferred into a petri dish. Up to three drops of methylene blue were added and distributed by swirling the petri dish gently. The sediment was examined at ×100 magnification.

### Data management

The laboratory data were entered into Microsoft Excel, version 2010. The biodata and questionnaire data were entered using Census and Survey Processing System, version 4.1. (US Census Bureau), and subsequently exported to Microsoft Excel, version 2010. The datasets were merged and prepared for data cleaning and descriptive and statistical analysis in STATA 13.1 (StataCorp).

### Statistical analysis

Results from the fecal analysis (presence/absence of eggs from any of the gastrointestinal helminths) were merged with the pig biodata and the questionnaire data (household characteristics including pig management variables). A dichotomous outcome variable was computed as the presence or absence of any of the gastrointestinal helminths in each pig. Descriptive statistics of all variables were computed to detect abnormal values. Tables 1 and 2 list all the variables evaluated. All subsequent analyses were restricted to pigs between 3 and 36 months of age.

**Table 1:** List of all predictors (including quadratic terms) relating to individual animals examined and individual pig-keeping household characteristics, descriptions, and unconditional association (*p* value) with gastrointestinal helminth infection

Variables	Response choices	$\overline{x}$ ± *σ*_*x*_ (min–max)/*n* (%)	Odds ratio	*p* value	95 % CI
Individual animal variables					
Pigs aged 3–36 months	Overall	8.3 ± 4.8 (3–36)		≤0.001	
	Pig age centered		1.019	0.267	0.986–1.053
	Pig age squared		0.994	0.000	0.991–0.997
Pigs’ body weight (enumerator estimate)	Overall	40.3 ± 26.5 (5–220)		0.007	
	Pigs’ body weight centered		1.009	0.003	1.003–1.016
	Pigs’ body weight squared		≤1.000	0.030	≤1.000–1.000
Pig breed	overall			0.378	
	1 = local	157 (17.4)			
	2 = exotic	200 (22.2)	0.856	0.543	0.519–1.412
	3 = cross	515 (57.2)	0.755	0.184	0.449–1.143
Time elapsed since date of last deworming to date of sampling (days)	Overall	85.8 ± 84.1 (2–510)		0.063	
	Delta treatment centered		1.004	0.020	1.001–1.007
	Delta treatment squared		≤1.000	0.046	0.999–≤1.000
Individual pig-farming household variables					
Age of the pig farmer	Overall	46.8 ± 13.8 (15–99)		0.365	
	Age pig farmer centered		0.997	0.549	0.987–1.007
	Age pig farmer squared		1	0.201	≤1.000–1.001
Sex of the pig farmer	Overall			0.163	
	1 = male	606 (67.3)			
	2 = female	284 (31.5)	1.249	0.163	0.914–1.705
Education level of the pig farmer	Overall			0.303	
	1 = none	64 (7.1)			
	2 = primary	449 (49.8)	1.162	0.599	0.665–2.030
	3 = secondary	286 (31.7)	1.197	0.542	0.673–2.129
	4 = tertiary	70 (7.8)	0.713	0.365	0.343–1.481
	5 = other	11 (1.2)	0.553	0.384	0.146–2.099
One of the household’s major IGA, crop farming	Yes = 1; no = 0	695 (77.1)	1.153	0.473	0.781–1.703
One of the household’s major IGAs, animal keeping (including sales)	Yes = 1; no = 0	659 (73.1)	1.164	0.398	0.818–1.657
One of the household’s major IGAs, trading animal products (not own)	Yes = 1; no = 0	9 (1.0)	2.686	0.239	0.518–13.920
One of the household’s major IGAs, trading in agricultural products (not own produce)	Yes = 1; no = 0	20 (2.2)	0.487	0.123	0.195–1.216
One of the household’s major IGAs, formal salaried employee	Yes = 1; no = 0	89 (9.9)	0.770	0.272	0.482–1.228
One of the household’s major IGAs, business non-agricultural	Yes = 1; no = 0	243 (27.0)	1.007	0.363	0.993–1.021

*CI* confidence interval, *IGA* income-generating activity

**Table 2:** List of all predictors (including quadratic terms) relating to self-reported pig husbandry practices, descriptions, and unconditional association (*p* value) with gastrointestinal helminth infection

Variables	Response choices	$\overline{x}$ ± *σ*_*x*_(min–max)/*n* (%)	Odds ratio	*p* value	95 % CI
Frequency of pig deworming	Overall			0.431	
	0 = never	50 (5.6)			
	1 = monthly	268 (29.7)	0.748	0.389	0.387–1.448
	2 = quarterly	425 (47.2)	0.750	0.386	0.391–1.438
	3 = other	128 (14.2)	1.029	0.938	0.502–2.107
Dewormer used	Overall			0.246	
	1 = albendazole	257 (28.5)			
	2 = levamisol	102 (11.3)	0.802	0.496	0.425–1.514
	3 = ivermectin	356 (39.5)	1.061	0.814	0.649–1.733
	4 = piperazine	107 (11.9)	0.955	0.875	0.540–1.691
	5 = other	149 (16.5)	1.413	0.133	0.900–2.218
Herd size per pig farm (including piglets)		4.0 ± 3.8 (1–30)	0.982	0.427	0.938–1.027
Value chain type (production–consumption)	Overall			0.969	
	1 = rural-rural	603 (66.9)			
	2 = rural-urban	220 (24.4)	0.931	0.823	0.499–1.739
	3 = periurban-urban	78 (8.7)	0.925	0.875	0.349–2.450
Level of confinement	Overall			0.058	
	1 = tethered	418 (46.4)			
	2 = fully confined	387 (43.0)	0.728	0.078	0.511–1.036
	3 = other (free range, mixed)	88 (9.8)	0.600	0.041	0.368–0.980
Pigs feed on crop residues	Yes = 1; no = 0	883 (98.0)	1.271	0.764	0.266–6.070
Pigs feed on swill	Yes = 1; no = 0	292 (32.4)	0.949	0.753	0.683–1.318
Pigs feed on commercial feed products	Yes = 1; no = 0	537 (59.6)	0.939	0.720	0.666–1.324
Pigs feed on pastures	Yes = 1; no = 0	622 (69.0)	0.951	0.750	0.697–1.297
Where pig feeds are stored	Overall			0.332	
	1 = inside	612 (67.9)			
	2 = outside	133 (14.8)	1.140	0.608	0.691–1.881
	3 = other (not stored, mixed)	66 (7.3)	1.305	0.140	0.916–1.859
Farmers routinely quarantine new pigs	Yes = 1; no = 0	287 (31.8)	0.921	0.613	0.670–1.267
Farmers routinely practice terminal cleaning	Yes = 1; no = 0	249 (27.6)	0.788	0.153	0.568–1.093
Farmers practice routine cleaning	Yes = 1; no = 0	460 (51.0)	0.841	0.268	0.619–1.143
Routine cleaning and disinfecting of drinkers and feeders	Yes = 1; no = 0	330 (36.6)	1.009	0.957	0.737–1.381
Routinely wash and disinfect equipment and tools	Yes = 1; no = 0	269 (29.9)	0.927	0.646	0.671–1.282
Routinely remove manure and litter from the pens	Yes = 1; no = 0	677 (75.1)	0.620	0.009	0.434–0.885
Routinely use disinfectants	Yes = 1; no = 0	94 (10.4)	0.543	0.010	0.341–0.862
Do not mix pigs of different ages	Yes = 1; no = 0	517 (57.4)	0.985	0.921	0.734–1.322
Farmers change rubber boots	Yes = 1; no = 0	106 (11.8)	0.995	0.982	0.635–1.557
Farmers isolate sick pigs	Yes = 1; no = 0	565 (62.7)	0.878	0.411	0.645–1.197
Farmers consult a vet when the pig is sick	Yes = 1; no = 0	788 (87.5)	0.785	0.302	0.496–1.242
Farmers perform pest/rodent control	Yes = 1; no = 0	473 (52.5)	1.004	0.982	0.740–1.361

*CI* confidence interval

Univariable analysis between pig characteristics (e.g., pig age), household characteristics (e.g., age of farmer), pig management practices (e.g., routine manure removal from pens), and the outcome of interest (e.g., presence/absence of helminth eggs) were computed using a random effect logistic regression model with village as random effects. For continuous predictors, the linearity of the association was evaluated using Lowess curves (on the logit scale) and by adding quadratic terms to the model as needed. Variables with *p* < 0.15 were retained for multivariable modeling.

**Fig. 2:**
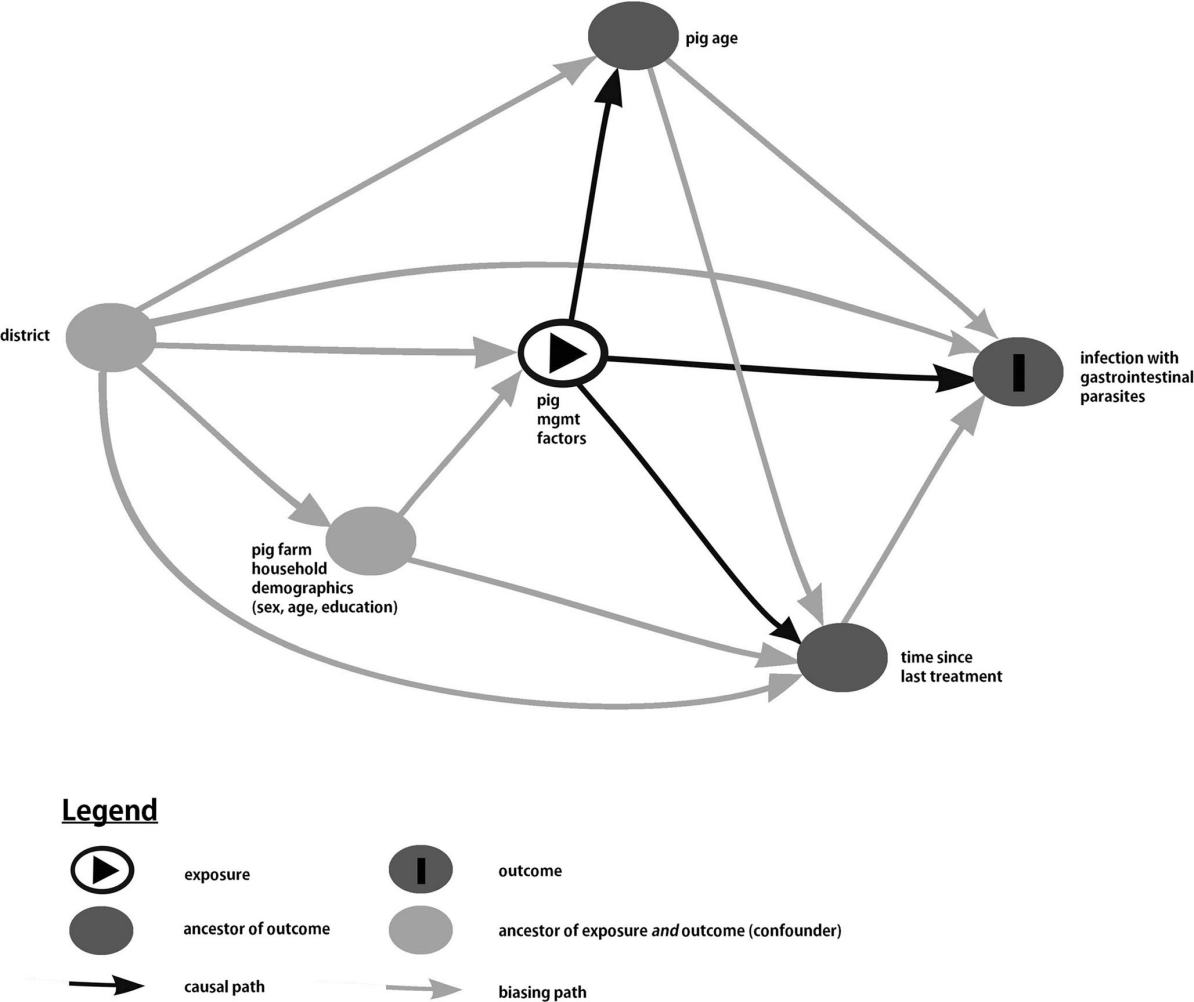
Causal diagram generated in DAGitty (Textor et al. 2011) postulating the relationships among the potential predictors and infection with intestinal parasites in pigs sampled in Central and Eastern Uganda between April and July 2013. A causal diagram, also known as directed acyclic graph (DAG), lays out the hypothesized causal relationships between variables with the direction of the *arrows* indicating the possible causal relationship. For example, pig management factors might influence pig age, but pig age does not change the management factors in place on the farm. When analyzing the effects of pig management factors, it is essential to control confounding factors which are those that are antecedent to (i.e., to the left of) pig management (e.g., district). Equally, it is important to not include intervening variables which are those between management factors and the outcome (e.g., pig age or time since last treatment). Inclusion of intervening variables results in the estimation of the “direct” effect of management practices and ignores indirect effects which are mediated through pig age or time since last treatment. For a more complete description of the use of causal diagrams, see Dohoo et al. (2009)

Given that there were, on average, only 1.6 villages per sub-county and that the between sub-county variance was either zero or very much smaller than the between village variance, multivariable modeling was carried out with village as the sole random effect. A causal diagram, or directed acyclic graph (DAG), was generated in the browser-based environment DAGitty® (Textor et al. 2011) to identify the relationships among the potential predictors (Fig. 2). Based on the causal model, three separate multivariable models were built with the main factor(s) of interest related to infection with gastrointestinal parasites being management factors, pig age, and time since last treatment. Variables antecedent to these factors (i.e., to the left in the causal diagram) were retained as potential confounders. Variables to the right of the factor(s) of interest were excluded from the model as they were intervening variables. Selection of variables to remain in the model was based on both statistical significance and potential confounding role (e.g., management effects were evaluated as possible confounders of pig age).

Regression diagnostics included examination of pig-level residuals and village random effects for extreme values. Normality and heteroscedasticity of village-level random effects were evaluated graphically.

## Results

In total, 1025 fecal samples were obtained in the multipathogen assessment between April and July 2013. If the related set of metadata was not complete, the animal was dropped from the analysis; this was the case for 99 animals. After another cleaning step, 901 animals and related metadata were included in the analyses, 299 from Kamuli District, 277 from Masaka District, and 325 from Mukono District.

### Prevalence of intestinal parasite eggs

Overall, 61.4 % of all animals tested positive for one or more gastrointestinal helminths, namely, strongyles, *Metastrongylus* spp., *Ascaris suum*, *Strongyloides ransomi*, and *Trichuris suis*; coccidia oocysts were found in 40.7 % of all pigs sampled. While all animals tested negative for *Fasciola* spp. and *B. coli*, 38.6 % were not infected with either helminths or coccidia. Details, including statistically significant differences across the districts, are presented in Table 3.

**Table 3:** Prevalence estimates of gastrointestinal parasites in smallholder pig production systems in Kamuli, Masaka, and Mukono Districts of Central and Eastern Uganda (April–July 2013)

District	Prevalence estimates (%) (calculated at *p* = 0.05, CI = 0.95)						
	Strongyles^a^	*Ascaris suum*	*Metastrongylus* spp.	*Strongyloides ransomi*	*Trichuris suis*	Any helminth infection	Coccidia^b^ oocysts
Kamuli	59.4 (53.7, 64.8)	11.2 (8.1, 15.3)	13.9 (10.4, 18.2)	1.0 (0.3, 3.0)	5.0 (3.0, 8.1)	66.0 (60.5, 71.1)	33.7 (28.6, 39.2)
Masaka	55.8 (49.9, 61.5)	3.6 (1.9, 6.6)	2.5 (1.2, 5.2)	4.0 (2.2, 7.0)	1.4 (0.5, 3.8)	57.6 (51.7, 63.3)	36.7 (31.2, 42.6)
Mukono	56.0 (50.6, 61.4)	2.8 (1.5, 5.3)	6.2 (4.0, 9.4)	7.4 (5.0, 10.9)	3.7 (2.1, 6.4)	60.4 (54.9, 65.6)	50.8 (45.3, 56.2)
Total	57.1 (53.8, 60.3)^#^	5.9 (4.5, 7.6)**	7.6 (6.1, 9.6)**	4.2 (3.1, 5.7)***	3.4 (2.4, 4.8)^#^	61.4 (58.2, 64.5)^#^	40.7 (37.5, 44.0)*

*CI* confidence interval

*p < 0.05

**p < 0.01

^#^not significant

^a^Strongyle eggs: *Oesophagostomum* spp., *Hyostrongylus rubidus*, and *Trichostrongylus axei*

^b^ *Eimeria* spp. and *Isospora suis*

### Descriptive analysis

Pigs sampled from all districts were about the same age, on average 8.3 months old, and weighed on average 45.9 kg. More than 40 % of the pig farmers claimed that they had treated their pigs with anthelminthics prior to sampling; however, the time since last treatment was on average 78.2 days, i.e., more than 11 weeks. While only 23.4 % of pig farmers in Mukono District and 41.3 % in Masaka District reported treatment of pigs with anthelminthic drugs, the proportion in Kamuli District was highest at 66.9 %. None of the famers reported treatment with coccidiostats or vaccination of any kind.

Tables 1 and 2 summarize the household demographics, pig herd parameters, and pig husbandry practices in the survey area. Overall, the majority of pigs were tethered, e.g., tied to a tree or pole with a rope on one of the limbs. Tethering was the predominant type of confinement in Kamuli District but less common in Mukono District and least so in Masaka District (68.2, 44.3, and 25.3 %, respectively). Full confinement or complete housing was mostly practiced in Masaka District (66.8 %), less so in Mukono District (38.5 %), and least so in Kamuli District (25.8 %). Only two pigs were exclusively kept free-ranging, one in Masaka and one in Mukono District. Other confinement types include seasonal mixing of free-ranging, tethering, and/or full housing, usually depending on the crop season (growing or harvesting).

Pigs in this survey were mostly fed on crop residues (i.e., sweet potato vines and tubers), pasture, commercial feeds (i.e., maize bran and dried fish), or swill (i.e., kitchen waste and bread). Other feeds included fruit grown and harvested locally. This cohort of pig farmers reported to routinely treat all pigs on the farm against gastrointestinal worms; however, the frequency differed from monthly in Kamuli District to quarterly in Masaka and Kamuli Districts. Routine practices performed by the pig farmers were cleaning of pig pens, removal of manure and litter, not mixing pigs of different age groups, isolating (clinically) sick pigs from healthy ones, consulting an animal health professional when pigs were ill, and routine pest and rodent control. Details of the descriptive statistics, including disaggregation by district, are presented in supplementary materials (S1–S4).

### Risk factors associated with gastrointestinal helminth infection

Four variables related to the individual animal examined, 9 variables related to the pig-farming household demographics, and 22 variables related to self-reported pig management practices were included in univariable analysis (Tables 1 and 2). Direct and indirect causal associations were postulated in a causal diagram (Fig. 2), which illustrates that pig management practices as a group (e.g., housing, feeding, routine cleaning and disinfection, and other biosecurity practices) are direct exposure variables associated with intestinal parasite infection. Both district and the sex of the household head were assumed to be potentially confounding variables, both associated with the explanatory variable but not a consequence of exposure to it. Pig age and time since last treatment were intervening variables and hence excluded from the model of management factors, while time since last treatment was excluded from the model of the effects of pig age.

Seven variables with *p* ≤ 0.15 were included in the multivariable analyses which retained four variables in the final three regression models (Table 4). District and sex of the household head were not statistically significant in the univariable analyses but forced into the models as potential confounders. Management factors that negatively correlated with gastrointestinal helminth infection were the routine removal of manure and litter from the pig pens (*p* ≤ 0.05, odds ratio [OR] = 0.667) and the routine use of disinfectants (*p* ≤ 0.05, OR = 0.548). In the model evaluating the effect of pig age (with district, sex of the household head, and the significant management factors included as confounders), the effect of pig age was not linear, so the highly significant quadratic term was retained. The quadratic relationship showed the prevalence of gastrointestinal helminths rising from 3 to 18 months of age and then falling up to 36 months of age. In the model evaluating the effect of time since last treatment (with district, sex of household head, management factors, and pig age included as confounders), there was no significant effect of time since last treatment. Three villages (Kijjabwemi, Kitete, and Kisoso) with the lowest prevalence (30–32 %) had large negative random effects. Removing these three villages from the model reduced the estimate of the between-village variation to zero and substantially reduced the power to detect effects of manure and disinfectant. Consequently, to avoid loss of information, they were retained in the analyses.

**Table 4:** Final models of multivariable logistic regression analysis for risk factors associated with gastrointestinal helminth infection in pigs in Central and Eastern Uganda

Model	Confounders controlled	Variable	Odds ratio	*p* value	95 % confidence interval
Effect of management practices	District, sex of head of household	Routinely remove manure and litter from the pens	0.667	0.029	0.464–0.959
		Routinely use disinfectants	0.548	0.013	0.340–0.882
Effect of age of pigs	District, sex of head of household, manure removal, use of disinfectants	Pig age centered	1.016	0.359	0.982–1.050
		Pig age squared	0.994	≤0.001	0.991–0.997
Effect of time since last treatment with antihelminthics	District, sex of head of household, manure removal, use of disinfectants, age of pig	Delta treatment centered	1.021	0.848	0.827–1.261

## Discussion

### Prevalence of intestinal helminths and coccidia

According to the results, infection with gastrointestinal parasites was common in pigs kept under smallholder conditions in Central and Eastern Uganda. Helminth eggs were found in more than half of the pigs; infections with strongyles and coccidia oocysts were most common in all study sites. This is consistent with other studies conducted in Uganda (Waiswa et al. 2007; Nissen et al. 2011), although those reported higher overall prevalence rates around 90 %; especially, levels of infection with *Metastrongylus* spp. and *A. suum* were much higher. This was also the case in studies from Kenya and Tanzania, where overall prevalence of one or more infections with helminths was similar to the present survey but levels of individual species such as *T. suis* and *A. suum* were higher (Esrony et al. 1997; Nganga et al. 2008). Discrepancy in overall infection rates may be caused by a different animal sampling approach; e.g., while we sampled one animal per farm (herd prevalence), Waiswa et al. (2007) examined all animals on one farm with one to three pigs and half of the animals on farms with more than four animals (animal prevalence). Other factors influencing prevalence could have been the different timing of the sampling (end of dry season), geography of the site (tropical highlands), and only growers (3–12 months of age) included, as in a study in Western Uganda (Nissen et al. 2011).

### Risk factors

The most important factors associated with gastrointestinal helminths in this cohort of pigs were pig age and routine husbandry practices related to sanitation, especially routine removal of manure and litter from the pig pens and the routine use of disinfectants. These results are consistent with findings from surveys in Kenya, where pigs are reared under similar settings (Kagira et al. 2008; Obonyo et al. 2013). Young pigs, especially suckling piglets and weaners, are usually most affected by parasitic infection and develop clinical signs, while in pigs older than 5 months, an age-related resistance (immunity) builds up, clinical signs are rare, and the number of helminth eggs shed decreases, except for strongyle *Oesophagostomum* spp. that accumulates with age (Roepstorff et al. 2011). A study conducted in the tropical highlands of Tanzania found significantly higher levels of helminth infections in local pigs than in the cross-bred animals kept in the semi-arid areas of the country (Esrony et al. 1997); however, this may have been related to the climate (temperature and precipitation) rather than the pig breed. In our study, all sites were located in the lowlands and pig breed was not significantly associated with the presence of helminth infection.

While a survey conducted in Nigeria by Weka and Ikeh (2009) found a negative correlation between the prevalence of intestinal parasites and routine deworming of the pigs, we found that administering anthelminthic drugs had no significant impact on the prevalence, even considering specific timing of deworming. However, our study recorded self-reported practices by farmers and we were not able to capture if the correct drugs were administered at the correct dosage. The most effective protective factors were the routine removal of manure and litter from pig pens and the use of disinfectants. Due to the pigs’ behavior of coprophagia, they are likely to ingest helminth eggs if feces are not regularly removed (Boes et al. 1997; Boes et al. 1998). Confinement or housing has previously been considered as a protective factor in Western Kenya, in a similar production setting (Kagira et al. 2012). In our study, it was not significantly associated with a lower prevalence; however, the study in Kenya used egg counts per gram as the outcome variable, while we evaluated presence or absence of helminth eggs. Moreover, we were not able to evaluate the effect of free-ranging because only two pigs in the cohort were kept exclusively free-ranging. The small number is likely to be due to the timing of the sampling, which took place at the end of the seasonal rains when crops had already been planted and pigs were confined to prevent them from damaging the growing plants.

### Potential economic implications

Infections with gastrointestinal parasites are more severe in piglets than in growers and adult pigs that comprised the sampled cohort in this study. Here, infections with gastrointestinal parasites are mostly sub-clinical; however, they can increase susceptibility to other endemic pathogens (Greve 2012). Monetary losses due to gastrointestinal parasite infection are very difficult to quantify. In Western countries, they are usually related to condemnation at slaughter (e.g., liver due to lesions caused by *A. suum*) and well documented. In Uganda, condemnation at slaughter is likely to play a secondary role as systematic carcass inspection and condemnation are not routinely practiced. Losses are likely to occur at the farm and to the farmers themselves and may be mostly related to losses in live weight gain due to reduced ADG and increased feed-to-gain ratios, potentially caused by gastric and/or intestinal ulcerations and, consequently, poor nutrient absorption. Farmers have to feed pigs for a longer time before they are market ready, which adds to the cost of raising them, especially feeds. Generally, faster growth rates result in higher daily feeding costs but greater revenue per pig; when pigs experience slow growth, the cumulative cost of feeding is much higher (Levy et al. 2014).

Experiments have shown that growing pigs infected with one or more species, in particular *A. suum*, *T. suis*, or *Oesophagostomum* spp., experience reduced growth rates of 33 % due to poor food utilization (Hale and Stewart 1979; Hale et al. 1985) and changed body composition, e.g., heavier plucks and less meat (reviewed by Roepstorff et al. 2011). Experiments under field conditions showed that losses in ADG were significant in pigs with heavy and longer-duration ascarid infections (Bernardo et al. 1990). In other studies, heavy ascarid burden decreased ADG, feed conversion, and lean meat percentage, but the effect was not significant (Boes et al. 2010). These experiments were conducted in industrialized countries with potentially improved pigs and a much more controlled environment (e.g., otherwise balanced diets and vaccination), and to the authors’ knowledge, there are no comparable experimental data available for local pig breeds infected with any of the helminths studied from tropical smallholder production systems.

### Target interventions: prevention versus treatment?

Group discussions with farmers in the study sites showed that parasites are considered to be a cause of poor performance and that treatment is mostly curative and not based on diagnosis (Dione et al. 2014). More than 90 % of the farmers claimed that they deworm their pigs at least once, shortly before sale or slaughter, mostly by means of an ivermectin injection (1–1.80 US dollars). The investment in the ivermectin injection is likely to result in higher returns if piglets receive preventive treatment when they are still very young in order to keep the parasite burden low and help the animals build up immunity. Generally, routine preventive treatment of pigs on organic farms is considered not an option (Krecek and Waller 2006; Nissen et al. 2011; Roepstorff et al. 2011), and the traditional pig sector in the tropics and sub-tropics can be greatly improved by relatively modest inputs such as good sanitation and basic housing (Verhulst 1993; Lekule and Kyvsgaard 2003; Krecek and Waller 2006). Our study supports the argument that expensive treatment cannot be the only method for controlling parasites if good hygienic practices such as regular removal of feces and the use of disinfectant are not applied.

### Limitations of the study

The present study was part of a multipathogen assessment with the main objective to systematically identify pig diseases prevalent in smallholder production systems in Central and Eastern Uganda. We therefore abstained from species identification through oocyst sporulation and larval migration as we did not see added value to this in answering the research questions for this study. In future, the disease burden and effectiveness of interventions should be quantified in longitudinal studies and by means of egg counts. One shortcoming of the study is that it did not include pigs younger than 3 months, which are most prone to parasitic infections and associated impacts.

## Conclusion

This study showed that infection with gastrointestinal parasites is common in pigs kept under smallholder conditions in Central and Eastern Uganda. Almost two thirds of the pigs were infected with one or more of the parasites studied, predominantly strongyles followed by coccidia. The most significant risk factors identified are relatively easy to control at the individual farm level. These were routine removal of manure and litter and routine disinfection to manage gastrointestinal helminths.

These biosecurity-related practices may be effective not only against parasites that limit productivity but also against other highly infectious pathogens such as African swine fever virus and other endemic pathogens. The additional monetary value of interventions is difficult to quantify, and more experimental and field research are needed on the cost and impact of single or collective interventions to improve husbandry practices in the local settings.

## Electronic supplementary material

Below is the link to the electronic supplementary material.

ESM 1 (DOCX 45.7 kb)

## Footnotes

1 Based on authors’ calculations, pig numbers were retrieved from the 2008 National Livestock Census (MAAIF/UBOS 2009) and data on district areas were obtained from the Uganda Districts Information Handbook, expanded edition 2011–2012 by Fountain Publishers, Kampala, Uganda.
