# Supplementary material for: Prevalence and risk factors for gastrointestinal parasites in small-scale pig enterprises in Central and Eastern Uganda
Source: Parasitol Res. 2016 Oct 26;116(1):335–45. doi: 10.1007/s00436-016-5296-7 (PMC5167772; doi:10.1007/s00436-016-5296-7)
Supplement: Supplementary file 1 — (DOCX 45.7 kb) [file 436_2016_5296_MOESM1_ESM.docx]

Title: Prevalence and risk factors for gastrointestinal parasites in small-scale pig enterprises in Central and Eastern Uganda.

Journal Name: Parasitology Research

Authors: Kristina Roesel, Ian Dohoo, Maximilian Baumann, Michel Dione, Delia Grace, Peter-Henning Clausen

Corresponding author affiliation and e-mail address:

Freie Universität Berlin, Institute for Parasitology and Tropical Veterinary Medicine, Robert-von-Ostertag-Str. 7-13, 14163 Berlin, Germany

International Livestock Research Institute, P.O. Box 30907, Nairobi 00100, Kenya

k.roesel@cgiar.org

Table S1: Prevalence estimates gastrointestinal parasites in 21 villages in Kamuli, Masaka and Mukono districts of Central and Eastern Uganda (April-July 2013)

| **District** | **Village** | **Prevalence estimates (%)**  **(calculated at p = 0.05, CI = 0.95)** | | | | | | |
| --- | --- | --- | --- | --- | --- | --- | --- | --- |
|  |  | **Strongyles^a^** | ***Ascaris suum*** | ***Metastrongylus* spp.** | ***Strongyloides ransomi*** | ***Trichuris suis*** | **Any helminth infection** | **Coccidia^b^ oocysts** |
|  | Butabaala | 66.7 (46.9; 81.9) | 7.4 (1.8; 25.8) | 11.1 (3.5; 29.9) | 0.0 | 11.1 (3.5; 29.9) | 77.8 (58.1; 89.8) | 22.2 (10.2; 41.9) |
|  | Bukyonza B | 66.7 (46.9; 81.9) | 18.5 (7.8; 38.0) | 11.1 (3.5; 29.9) | 7.4 (1.8; 25.8) | 7.4 (1.8; 25.8) | 70.4 (50.6; 84.7) | 37.0 (21.0; 56.6) |
|  | Baluboinewa | 67.5 (51.5; 80.3) | 17.5 (8.5; 32.7) | 5.0 (1.2; 18.2) | 0.0 | 0.0 | 70.0 (54.0; 82.3) | 52.5 (37.1; 67.5) |
|  | Isingo A | 44.4 (31.7; 57.9) | 9.3 (3.9; 20.6) | 9.3 (3.9; 20.6) | 1.9 (0.3; 12.2) | 5.6 (1.8; 16.0) | 55.6 (42.1; 68.3) | 25.9 (15.9; 39.3) |
|  | Ntansi | 54.7 (43.3; 65.6) | 10.7 (5.4; 20.0) | 20.0 (12.4; 30.7) | 0.0 | 5.3 (2.0; 13.5) | 60.0 (48.5; 70.5) | 28.0 (19.0; 39.3) |
|  | Kantu zone | 65.0 (53.9; 74.7) | 8.8 (4.2; 17.3) | 17.5 (10.6; 27.5) | 0.0 | 3.4 (1.2; 11.1) | 71.3 (60.3; 80.2) | 37.5 (27.6; 48.6) |
| **Kamuli** |  | **59.4 (53.7; 64.8)** | **11.2 (8.1; 15.3)** | **13.9 (10.4; 18.2)** | **1.0 (0.3; 3.0)** | **5.0 (3.0; 8.1)** | **66.0 (60.5; 71.1)** | **33.7 (28.6; 39.2)** |
|  | Ssenyange A | 88.6 (72.9; 95.7) | 5.7 (1.4; 20.5) | 5.7 (1.4; 20.5) | 5.7 (1.4; 20.5) | 0.0 | 88.6 (72.9; 95.7) | 22.9 (11.7; 39.8) |
|  | Butego | 71.4 (48.6; 86.9) | 4.8 (0.6; 28.2) | 0.0 | 4.8 (0.6; 28.2) | 0.0 | 71.4 (48.6; 86.9) | 23.8 (10.0; 46.7) |
|  | Kijjabwemi | 29.3 (17.3; 45.0) | 4.9 (1.2; 17.8) | 4.9 (1.2; 17.8) | 2.4 (0.3; 15.8) | 0.0 | 31.7 (19.3; 47.5) | 39.0 (25.3; 54.7) |
|  | Kisoso | 30.0 (17.8; 46.0) | 0.0 | 0.0 | 2.5 (0.3; 16.1) | 0.0 | 30.0 (17.8; 46.0) | 20.0 (10.2; 35.4) |
|  | Ssenya | 60.0 (41.6; 75.9) | 0.0 | 0.0 | 6.7 (1.6; 23.5) | 0.0 | 60.0 (41.6; 70.5) | 33.3 (18.8; 52.0) |
|  | Kyamuyimbwa-Kikalala | 57.1 (38.3; 74.1) | 7.1 (1.7; 25.0) | 0.0 | 3.6 (0.5; 22.1) | 7.1 (1.7; 25.0) | 64.3 (45.0; 79.9) | 28.6 (14.8; 48.0) |
|  | Lukindu | 63.9 (47.0; 77.9) | 2.8 (0.4; 17.7) | 0.0 | 0.0 | 0.0 | 63.9 (47.0; 77.9) | 55.6 (39.1; 70.9) |
|  | Kanoni-Bukunda | 59.6 (45.0; 72.7) | 4.3 (1.1; 15.7) | 6.4 (2.1; 18.2) | 6.4 (2.1; 18.2) | 4.3 (1.1; 15.7) | 63.8 (49.1; 76.3) | 57.5 (42.9; 70.8) |
| **Masaka** |  | **55.8 (49.9; 61.5)** | **3.6 (1.9; 6.6)** | **2.5 (1.2; 5.2)** | **4.0 (2.2; 7.0)** | **1.4 (0.5; 3.8)** | **57.6 (51.7; 63.3)** | **36.7 (31.2; 42.6)** |
|  | Kazo-Kalagala | 68.9 (53.9; 80.8) | 2.2 (0.3; 14.5) | 0.0 | 13.3 (6.1; 26.9) | 11.1 (4.7; 24.3) | 73.3 (58.5; 84.3) | 46.7 (32.6; 61.3) |
|  | Bugoye-Kabira | 54.3 (39.8; 68.2) | 2.2 (0.3; 14.2) | 8.7 (3.3; 21.2) | 2.2 (0.3; 14.2) | 2.2 (3.0; 14.2) | 58.7 (44.0; 72.0) | 41.3 (28.0; 56.0) |
|  | Nsanja-Gonve | 61.5 (45.4; 75.5) | 0.0 | 0.0 | 2.6 (0.4; 16.5) | 5.1 (1.3; 18.6) | 64.1 (47.9; 77.6) | 33.3 (20.3; 49.6) |
|  | Dundu | 60.9 (46.1; 73.9) | 6.5 (2.1; 18.6) | 13.0 (5.9; 26.3) | 8.7 (3.2; 21.2) | 2.2 (0.3; 14.2) | 71.7 (57.0; 82.9) | 43.5 (30.0; 58.1) |
|  | Kyoga | 53.9 (38.1; 68.8) | 5.1 (1.3; 18.6) | 7.7 (2.5; 21.6) | 5.1 (1.3; 18.3) | 2.6 (0.4; 16.5) | 59.0 (42.9; 73.3) | 53.9 (38.1; 68.8) |
|  | Joggo | 62.7 (49.7; 74.1) | 3.4 (0.8; 12.7) | 5.1 (1.6; 14.8) | 17.0 (9.3; 28.9) | 1.7 (0.2; 11.3) | 64.4 (51.4; 75.6) | 59.3 (46.3; 71.1) |
|  | Kitete | 30.6 (19.3; 44.9) | 0.0 | 8.2 (3.1; 20.0) | 0.0 | 2.0 (0.3; 13.4) | 32.7 (21.0; 47.0) | 71.4 (57.2; 82.4) |
| **Mukono** |  | **56.0 (50.6; 61.4)** | **2.8 (1.5; 5.3)** | **6.2 (4.0; 9.4)** | **7.4 (5.0; 10.9)** | **3.7 (2.1; 6.4)** | **60.4 (54.9; 65.6)** | **50.8 (45.3; 56.2)** |
| ***Total*** |  | ***57.1 (53.8; 60.3)^0^*** | ***5.9 (4.5; 7.6)^**^*** | ***7.6 (6.1; 9.6)^**^*** | ***4.2 (3.1; 5.7)^**^*** | ***3.4 (2.4; 4.8)^0^*** | ***61.4 (58.2; 64.5)^0^*** | ***40.7 (37.5; 44.0)^*^*** |

^a^Strongyle eggs: *Oesophagostomum* spp., *Hyostrongylus rubidus*, *Trichostrongylus axei*; ^b^*Eimeria* spp., *Isospora suis*

Significance across districts: ^0^not significant, *p<0.05,**p<0.01

Table S2: Descriptive statistics and medical history of individual pigs sampled in Central and Eastern Kamuli, Uganda, between April-July 2013

| Variable | Kamuli district  (n=299) | Masaka district  (n=277) | Mukono district  (n=325) | Total  (n=901) |
| --- | --- | --- | --- | --- |
|  | $\bar{x}$ ± σ_x_ (min-max) | $\bar{x}$ ± σ_x_ (min-max) | $\bar{x}$ ± σ_x_ (min-max) | $\bar{x}$ ± σ_x_ (min-max) |
| *Pigs aged 3-36 months* | 8.5±4.8 (3-30) | 8.5±5.1 (3-36) | 7.9±4.5 (3-36) | 8.3±4.8 (3-36) |
| *Pigs‘ body weight (enumerator estimate)* | 39.8±22.8 (5-111) | 45.9±3.8 (5-220) | 37.0±23.4 (5-200) | 40.3±26.5 (5-220) |
|  | | | | |
| *Pig breed* | n (%) | n (%) | n (%) | n (%) |
| Local | 23 (7.7) | 64 (23.1) | 70 (21.5) | 157 (17.4) |
| Exotic | 126 (42.1) | 9 (3.3) | 65 (20.0) | 200 (22.2) |
| Cross | 141 (47.2) | 189 (68.2) | 185 (56.9) | 515 (57.2) |
| Missing | 9 (3.0) | 15 (5.4) | 5 (1.6) | 29 (3.2) |
|  |  |  |  |  |
| *Treated with antiparasitic drugs*  *prior to sampling* | n (%) | n (%) | n (%) | n (%) |
| Yes | 200 (66.9) | 114 (41.2%) | 76 (23.4%) | 390 (43.3) |
| No | 32 (10.7) | 0 | 9 (2.8 %) | 41 (4.6) |
| Missing | 67 (22.4) | 163 (58.8%) | 240 (73.8%) | 470 (52.2) |
|  |  |  |  |  |
|  | $\bar{x}$ ± σ_x_ (min-max) | $\bar{x}$ ± σ_x_ (min-max) | $\bar{x}$ ± σ_x_ (min-max) | $\bar{x}$ ± σ_x_ (min-max) |
| *Time elapsed since date of last deworming to date of sampling (days)* | 78.2±78.8 (2-510) | 120.7±113.1 (8-464) | 69.9±55.5 (2.5-441) | 85.8±84.1 (2-510) |
| *Time since pig last sick* | 77.1±67.9 (2-451) | 112.5±109.3 (1.5-463) | 61.2±47.1 (1-232) | 79.9±75.1 (1-463) |
|  |  |  |  |  |
| *Symptoms, if sick recently^c^*  *(multiple anwers)* | n (%) | n (%) | n (%) | n (%) |
| Diarrhea | 36 (12.0) | 19 (6.9) | 23 (7.1) | 78 (8.7) |
| Lack of appetite | 30 (10.0) | 18 (6.5) | 28 (8.6) | 76 (8.4) |
| Vomiting | 12 (4.0) | 6 (2.2) | 14 (4.3) | 32 (3.6) |
| Coughing | 35 (11.7) | 5 (1.8) | 17 (5.2) | 57 (6.3) |
| Missing | 177 (59.2) | 218 (78.7) | 258 (79.4) | 653 (72.5) |
|  |  |  |  |  |

^c^Other symptoms recorded but not presented here were dullness, fever, swaying gait, skin rashes, foaming mouth, shivering and wounds.

Table S3: Descriptive statistics on pig-farming house hold demographics in Central and Eastern Kamuli, Uganda (April-July 2013)

| Variable | Kamuli district  (n=299) | Masaka district  (n=277) | Mukono district  (n=325) | Total  (n=901) |
| --- | --- | --- | --- | --- |
|  | $\bar{x}$ ± σ_x_(min-max) | $\bar{x}$ ± σ_x_(min-max) | $\bar{x}$ ± σ_x_(min-max) | $\bar{x}$ ± σ_x_(min-max) |
| *Age of the pig farming house hold head* | 46.3 ± 13.1 (19-81) | 47.0 ± 14.8 (15-84) | 47.2 ± 13.4 (19-99) | 46.8 ± 13.8 (15-99) |
|  |  |  |  |  |
|  | n (%) | n (%) | n (%) | n (%) |
| *Sex of the pig farming household head* |  |  |  |  |
| Male | 209 (69.9) | 161 (58.1) | 236 (72.6) | 606 (67.3) |
| Female | 89 (29.8) | 112 (40.4) | 83 (25.5) | 284 (31.5) |
| Missing | 1 (0.3) | 4 (1.5) | 6 (1.9) | 11 (1.2) |
| *Religion* |  |  |  |  |
| Christian^d^ | 296 (99.0) | 273 (98.6) | 301 (92.6) | 870 (96.6) |
| Muslim | 0 | 2 (0.7) | 2 (0.6) | 4 (0.4) |
| Other (Traditional African Faith, Mormon) | 1 (0.3) | 0 | 1 (0.3) | 2 (0.2) |
| Missing | 2 (0.7) | 2 (0.7) | 21 (6.5) | 25 (2.8) |
| *Ethnic group* |  |  |  |  |
| Muganda | 0 | 251 (90.6) | 278 (85.5) | 529 (58.7) |
| Musoga | 281 (94.0) | 1 (0.4) | 9 (2.8) | 291 (32.3) |
| Other | 4 (0.3) | 23 (8.3) | 30 (9.2) | 57 (6.3) |
| Missing | 14 (4.7) | 2 (0.7) | 8 (2.5) | 24 (2.7) |
| *Education level* |  |  |  |  |
| None | 26 (8.7) | 12 (4.3) | 26 (8.0) | 64 (7.1) |
| Primary | 151 (50.5) | 152 (54.9) | 146 (44.9) | 449 (49.8) |
| Secondary | 101 (33.8) | 79 (28.5) | 106 (32.6) | 286 (31.7) |
| Tertiary | 17 (5.7) | 20 (7.2) | 33 (10.2) | 70 (7.8) |
| Other | 3 (1.0) | 5 (1.8) | 3 (0.9) | 11 (1.2) |
| Missing | 1 (0.3) | 9 (3.25) | 11 (3.4) | 21 (2.3) |
| *Major income-generating activities*  *(multiple answers)* |  |  |  |  |
| Crop farming | 242 (80.9) | 254 (91.7) | 199 (61.2) | 695 (77.1) |
| Animal keeping (incl. sales) | 214 (71.6) | 274 (98.9) | 171 (52.6) | 659 (73.1) |
| Trading animal products (not own) | 1 (0.3) | 6 (2.2) | 2 (0.6) | 9 (1.0) |
| Trading in agricultural products (not own produce) | 7 (2.3) | 8 (2.9) | 5 (1.5) | 20 (2.2) |
| Formal salaried employer | 30 (10.0) | 29 (10.5) | 30 (9.2) | 89 (9.9) |
| Business non-agricultural^e^ | 18 (6.0) | 118 (42.6) | 107 (32.9) | 243 (27.0) |
| *Routine self-deworming (pig farmer)* |  |  |  |  |
| Yes | 126 (42.1) | 177 (63.9) | 255 (78.5) | 558 (61.9) |
| No | 171 (57.2) | 96 (34.7) | 69 (21.2) | 336 (37.3) |
| Missing | 2 (0.7) | 4 (1.4) | 1 (0.3) | 7 (0.8) |
| Total | 299 (100.0) | 277 (100.0) | 325 (100.0) | 901 (100.0) |
| *Frequency of self-deworming (pig farmer)* |  |  |  |  |
| Never | 171 (57.2) | 96 (34.7) | 69 (21.2) | 336 (37.3) |
| Monthly | 37 (12.4) | 25 (9.0) | 18 (5.5) | 80 (8.9) |
| Quarterly | 38 (12.7) | 86 (31.1) | 149 (45.8) | 273 (30.3) |
| Other | 50 (16.7) | 65 (23.5) | 86 (26.5) | 201 (22.3) |
| Missing | 3 (1.0) | 5 (1.8) | 3 (0.9) | 11 (1.2) |

^d^Includes Catholics, Protestants, Adventists, Borne Again, Lutherans, Jehovas Witnesses

^e^includes kiosk owners, taxi drivers, mechanics, blacksmith, brick maker, tailors etc.

Table S4: Descriptive statistics on self-reported pig husbandry practices in pig-farming house holds in Central and Eastern Kamuli, Uganda (April-July 2013)

| Variable | Kamuli district  (n=299) | Masaka district  (n=277) | Mukono district  (n=325) | Total  (n=901) |
| --- | --- | --- | --- | --- |
| *Total herd size* | $\bar{x}$ ± σ_x_(min-max) | $\bar{x}$ ± σ_x_(min-max) | $\bar{x}$ ± σ_x_(min-max) | $\bar{x}$ ± σ_x_(min-max) |
|  | 3.3 ±3.2 (1-26) | 4.0±3.2 (1-15) | 5.1 ± 4.3 (1-30) | 4.0 ± 3.8 (1-30) |
|  |  |  |  |  |
|  | n (%) | n (%) | n (%) | n (%) |
| *Value chain types*  *(production – consumption)* |  |  |  |  |
| Rural-rural | 299 (100.0) | 65 (23.5) | 239 (73.5) | 603 (66.9) |
| Rural-urban | 0 | 134 (48.4) | 86 (26.5) | 220 (24.4) |
| Periurban-urban | 0 | 78 (28.9) | 0 | 78 (8.7) |
| *Level of confinement* |  |  |  |  |
| Tethered | 204 (68.2) | 70 (25.3) | 144(44.3%) | 418 (46.4) |
| Fully confined | 77 (25.8) | 185 (66.8) | 125 (38.5) | 387 (43.0) |
| Other^f^ | 16 (5.4) | 21 (7.6) | 51 (15.7) | 88 (9.8) |
| Missing | 2 (0.7) | 1 (0.4) | 5 (1.5) | 8 (0.9) |
| *Feed type (multiple answers)* |  |  |  |  |
| Crop | 297/299 (99.3) | 274/277 (98.9) | 312/325 (96.0) | 883/901 (98.0) |
| Swill | 4/299 (1.3) | 114/277 (41.2) | 174/325 (53.5) | 292/901 (32.4) |
| Commercial | 245/299 (81.9) | 137/277 (49.5) | 155/325 (47.7) | 537/901 (59.6) |
| Pasture | 213/299 (71.2) | 160/277 (57.8) | 249/325 (76.6) | 622/901 (69.0) |
| Others | 24/299 (8.0) | 88/277 (31.8) | 86/325 (26.5) | 208/901 (23.1) |
| Missing | 1/299 (0.3) | 1/277 (0.4) | 2/325 (0.6) | 4/901 (0.4) |
| *Feed store (multiple answers)* |  |  |  |  |
| Confined in a store | 220/299 (73.6) | 205/299 (74.0) | 187/299 (57.5) | 612 (67.9) |
| Outside in the garden | 31/299 (10.4) | 3/299 (1.1) | 99/299 (30.5) | 133 (14.8) |
| Missing | 37/299 (12.4) | 58/299 (20.9) | 18/299 (5.5) | 113 (12.5) |
| *Deworming of all pigs on farm routinely practiced* |  |  |  |  |
| Yes | 265 (88.6) | 264 (95.3) | 318 (97.9) | 847 (94.0) |
| No | 33 (11.1) | 13 (4.7) | 4 (1.2) | 50 (5.6) |
| Missing | 1 (0.3) | 0 | 3 (0.9) | 4 (0.4) |
| *Frequency of deworming* |  |  |  |  |
| Never | 33 (11.1) | 13 (4.7) | 4 (1.2) | 50 (5.6) |
| Monthly | 124 (41.5) | 62 (22.4) | 82 (25.2) | 268 (29.7) |
| Quarterly | 92 (30.1) | 142 (51.3) | 191 (58.8) | 425 (47.2) |
| Other | 43 (14.4) | 46 (16.6) | 39 (12.0) | 128 (14.2) |
| Missing | 7 (2.3) | 14 (5.1) | 9 (2.8) | 30 (3.3) |
| *Dewormer used (multiple answers)* |  |  |  |  |
| Albendazole | 130/299 (43.5) | 87/277 (31.4) | 40/325 (12.3) | 257/901 (28.5) |
| Levamisol | 0 | 12/277 (4.3) | 90/325 (27.7) | 102/901 (11.3) |
| Ivermectine | 11/299 (3.7) | 226/277 (81.6) | 119/325 (36.6) | 356/901 (39.5) |
| Piperazine | 106/299 (35.5) | 0 | 1/325 (0.3) | 107/901 (11.9) |
| Others (don’t know/ vet decides/ local herbs) | 63/299 (21.1) | 1/277 (0.4) | 85/325 (26.2) | 149/901 (16.5) |
| Missing | 35/299 (11.7) | 11/277 (4.0) | 16/325 (4.9) | 62/901 (6.9) |
| *Other animals kept on the farm*  *(multiple answers)* |  |  |  |  |
| Cows | 166/299 (55.5) | 76/277 (27.4) | 152/325 (46.8) | 394/901 (43.7) |
| Dogs | 38/299 (12.7) | 68/277 (24.6) | 130/325 (40.0) | 236/901 (26.2) |
| Goats | 167/299 (55.9) | 70/277 (25.3) | 122/325 (37.5) | 359/901 (39.8) |
| Poultry | 244/299 (81.6) | 215/277 (77.6) | 239/325 (73.5) | 698/901 (77.5) |
| Sheep | 0 | 3/277 (1.1) | 42/325 (12.9) | 45/901 (5.0) |
| Others (cats, rabbits etc.) | 4/299 (1.3) | 34/277 (12.3) | 16/325 (4.9) | 54/901 (6.0) |
| Missing | 18/299 (6.0) | 21/277 (7.6) | 39/325 (12.0) | 78/901 (8.7) |

^f^Includes two pigs that are exclusively free-ranging, 17 pigs that are seasonally free-roaming, and 69 that are kept mixed (seasonally partly or fully confined)

| Variable | Kamuli district  (n=299) | Masaka district  (n=277) | Mukono district  (n=325) | Total  (n=901) |
| --- | --- | --- | --- | --- |
| Routine quarantine of newly purchased pigs |  |  |  |  |
| Yes | 138 (46.2) | 99 (35.7) | 50 (15.4) | 287 (31.8) |
| No | 160 (53.5) | 159 (57.4) | 268 (82.5) | 587 (65.2) |
| Missing | 1 (0.3) | 19 (6.9) | 7 (2.1) | 27 (3.0) |
| Routine terminal cleaning  (end of cycle cleaning) |  |  |  |  |
| Yes | 65 (21.7) | 102 (36.8) | 82 (25.2) | 249 (27.6) |
| No | 233 (77.9) | 124 (44.8) | 224 (68.9) | 581 (64.5) |
| Missing | 1 (0.3) | 43 (15.5) | 19 (5.9) | 71 (7.9) |
| Routine cleaning (of pig pens) |  |  |  |  |
| Yes | 169 (56.5) | 143 (51.6) | 148 (45.5) | 460 (51.0) |
| No | 129 (43.2) | 91 (32.9) | 161 (49.5) | 381 (42.3) |
| Missing | 1 (0.3) | 43 (15.5) | 16 (4.9) | 60 (6.7) |
| Routine cleaning and disinfection of drinkers and feeders |  |  |  |  |
| Yes | 175 (58.5) | 82 (29.6) | 73 (22.5) | 330 (36.6) |
| No | 122 (40.8) | 182 (65.7) | 246 (75.7) | 550 (61.0) |
| Missing | 2 (0.7) | 13 (4.7) | 6 (1.8) | 21 (2.3) |
| Routine washing and disinfection of (farm) equipment and tools |  |  |  |  |
| Yes | 146 (48.8) | 78 (28.2) | 45 (13.9) | 269 (29.9) |
| No | 151 (50.5) | 192 (69.3) | 275 (84.6) | 618 (68.6) |
| Missing | 2 (0.7) | 7 (2.5) | 5 (1.5) | 14 (1.5) |
| Routine removal of manure and litter (from the pig pens) |  |  |  |  |
| Yes | 223 (74.6) | 203 (73.3) | 251 (77.2) | 677 (75.1) |
| No | 74 (24.8) | 68 (24.5) | 66 (20.3) | 208 (23.1) |
| Missing | 2 (0.7) | 6 (2.2) | 8 (2.5) | 16 (1.8) |
| Routine use of disinfectants |  |  |  |  |
| Yes | 67 (22.4) | 6 (2.2) | 21 (6.5) | 94 (10.4) |
| No | 229 (76.6) | 266 (96.0) | 299 (92.0) | 794 (88.1) |
| Missing | 3 (1.0) | 5 (1.8) | 5 (1.5) | 13 (1.9) |
| Farmer does not usually mix pigs of different ages |  |  |  |  |
| Yes | 183 (61.2) | 139 (50.2) | 195 (60.0) | 517 (57.4) |
| No | 114 (38.1) | 125 (45.0) | 124 (38.2) | 363 (40.3) |
| Missing | 2 (0.7) | 13 (4.7) | 6 (1.8) | 21 (2.3) |
| Routine changing of rubber boots |  |  |  |  |
| Yes | 61 (20.4) | 38 (13.7) | 7 (2.2) | 106 (11.8) |
| No | 236 (78.9) | 231 (83.4) | 312 (96.0) | 779 (86.4) |
| Missing | 2 (0.7) | 8 (2.9) | 6 (1.8) | 16 (1.8) |
| Routine separation/isolation of sick pigs |  |  |  |  |
| Yes | 209 (69.9) | 127 (45.9) | 229 (70.5) | 565 (62.7) |
| No | 87 (29.1) | 133 (48.0) | 89 (27.4) | 309 (34.3) |
| Missing | 3 (1.0) | 17 (6.1) | 7 (2.1) | 27 (3.0) |
| Consultation of vet when pigs are sick |  |  |  |  |
| Yes | 265 (88.6) | 247 (89.2) | 276 (84.9) | 788 (87.5) |
| No | 32 (10.7) | 25 (9.0) | 42 (12.9) | 99 (11.0) |
| Missing | 2 (0.7) | 5 (1.8) | 7 (2.2) | 14 (1.5) |
| Routine pest and rodent control |  |  |  |  |
| Yes | 190 (63.6) | 72 (26.0) | 211 (64.9) | 473 (52.5) |
| No | 106 (35.5) | 198 (71.5) | 107 (32.9) | 411 (45.6) |
| Missing | 3 (1.0) | 7 (2.5) | 7 (2.2) | 17 (1.9) |
